# Supplementary material for: The Methyltransferase Region of Vesicular Stomatitis Virus L Polymerase Is a Target Site for Functional Intramolecular Insertion
Source: Viruses. 2019 Oct 26;11(11):989. doi: 10.3390/v11110989 (PMC6893670; doi:10.3390/v11110989)
Supplement: Supplementary file 1 [file viruses-11-00989-s001.zip › supplementary files/suppl files captions.docx]

**Figure S1. Sequences of VSV-L-mCherry genomes.** Virus genomic RNA was purified and reverse transcribed to cDNA, which became the template for PCRs on regions of interest. Two L-mCherry viruses were sequenced after generation (ag) of viruses by reverse genetics and after passaging (ap) of viruses. The plasmid sequence was aligned with these four genome sequences (mCherry 1/2 ag/ap). Sections of interests of the alignments are shown (mCherry and sites of mutations). Mutation-bearing sections are enlarged and shown in a separate frame. Mutations of nucleotide and resulting amino-acid sequences are highlighted.

**Figure S2. Sequences of VSV-L-mWasabi genomes.** Virus genomic RNA was purified and reverse transcribed to cDNA, which became the template for PCRs on regions of interest. Two L-mWasabi viruses were sequenced after generation of viruses by reverse genetics. The plasmid sequence was aligned with these two genome sequences. Sections of interests of the alignments are shown (sites of mutations). Mutation-bearing sections are enlarged and marked by separate frames. Mutations of nucleotide and resulting amino-acid sequences are highlighted.

**Video S1. High magnification fluorescence microscopy movie underlines distinctive fluorescence pattern of GFP and L-mCherry.** Assessment of the kinetic of GFP vs. mCherry fluorescence in time-lapse recording: BHK-21 cells were infected with VSV-GFP-L-mCherry at MOI of 10 at 37°C. Images were acquired for 12 hours after infection. A movie was generated from 10-minute interval images of one representative cell in phase contrast (left), TRITC (middle) and FITC (right) channels.
