# Supplementary figures and images for: The Methyltransferase Region of Vesicular Stomatitis Virus L Polymerase Is a Target Site for Functional Intramolecular Insertion
Source: Viruses. 2019 Oct 26;11(11):989. doi: 10.3390/v11110989 (PMC6893670; doi:10.3390/v11110989)

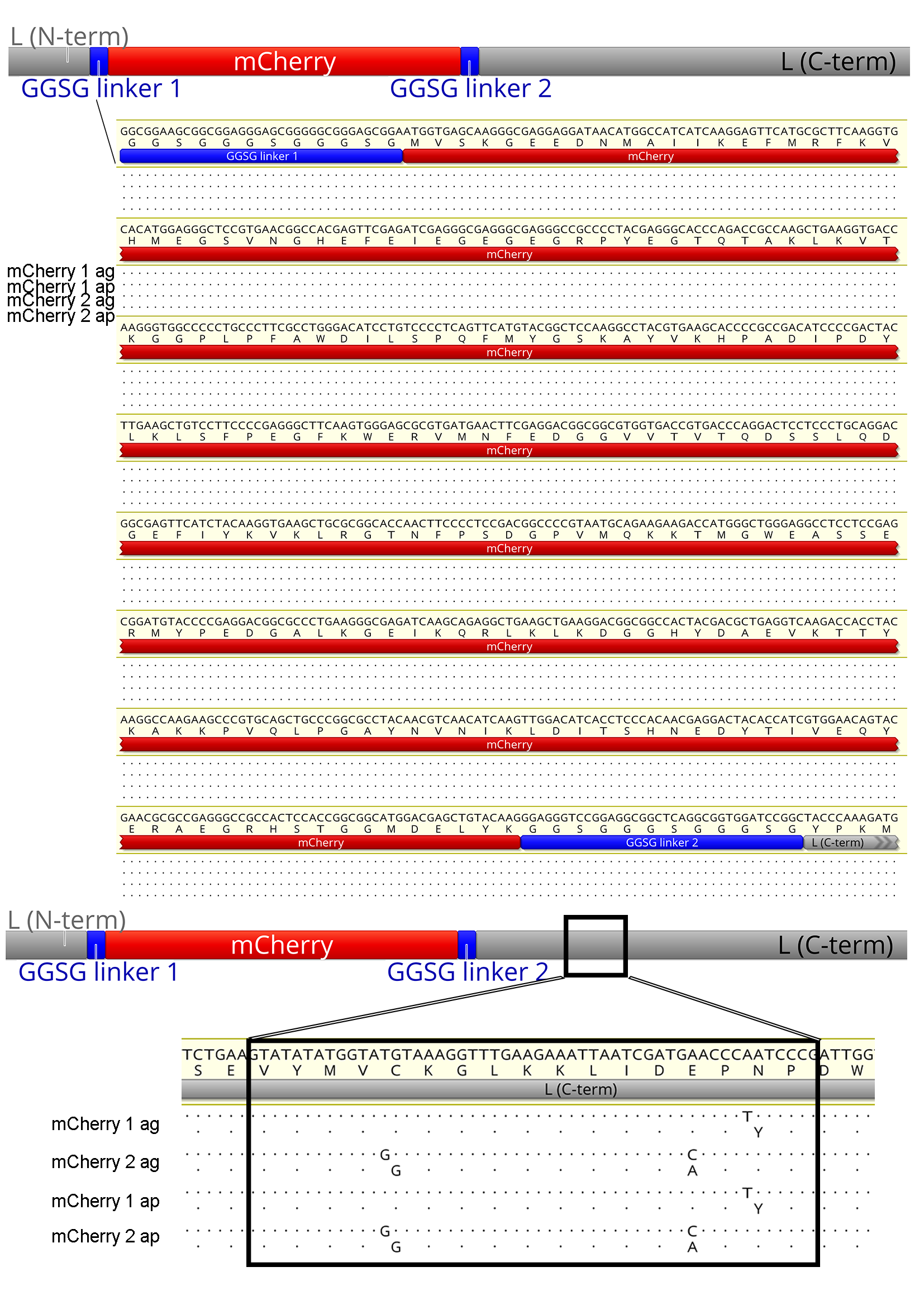

Supplement: Supplementary file 1 [file viruses-11-00989-s001.zip › supplementary files/suppl figure S1 revised.tif]

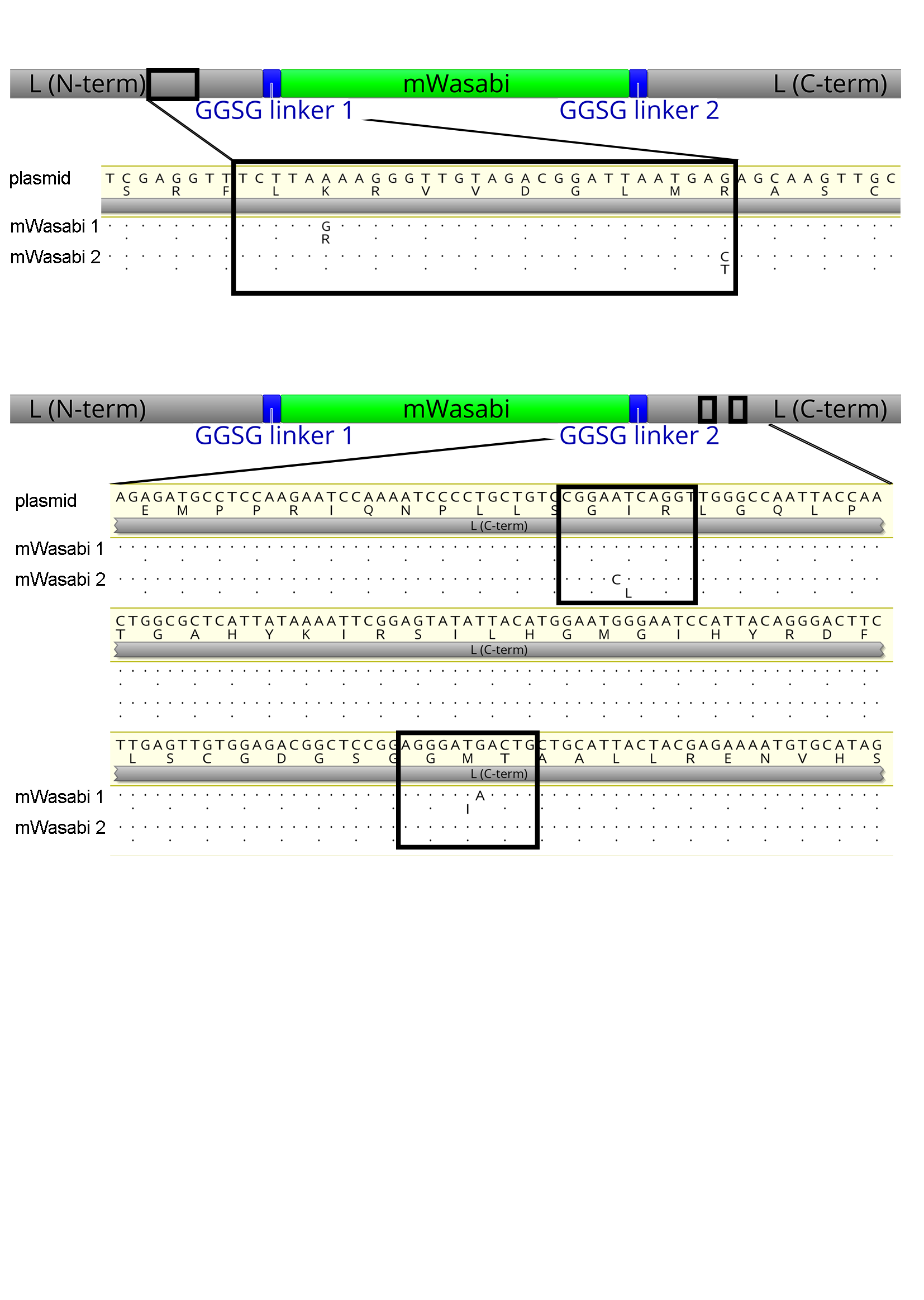

Supplement: Supplementary file 1 [file viruses-11-00989-s001.zip › supplementary files/suppl figure S2.tif]
